# Transcranial stimulation of the frontal lobes increases propensity of mind-wandering without changing meta-awareness

**DOI:** 10.1038/s41598-018-34098-z

**Published:** 2018-10-29

**Authors:** Vadim Axelrod, Xingxing Zhu, Jiang Qiu

**Affiliations:** 1 The Gonda Multidisciplinary Brain Research Center, Bar Ilan University, Ramat Gan, 52900 Israel; 2 School of Psychology, Southwest University, Chongqing, 400715 China; 3 Key Laboratory of Cognition and Personality of Ministry of Education, Southwest University, Chongqing, 400715 China

## Abstract

Mind-wandering is omnipresent in our lives. The benefits of mind-wandering are not yet clear, but given how much time we spend mind-wandering, this mental function is likely to be important. Accordingly, it is essential to understand the neural and cognitive mechanisms of mind-wandering. In a recent study by the leading author of the present paper it was demonstrated that by applying transcranial direct current stimulation (tDCS) of the frontal lobes, but not sham or occipital cortex stimulation, it was possible to increase propensity of mind-wandering. The goal of the present study has been to replicate these previous findings and to extend them by examining whether changes in mind-wandering as a result of stimulation are associated with a change of meta-awareness of the attentional focus. By using a larger sample size and by conducting the experiment in a different country and language, we fully replicated the key original findings by showing that stimulation of the prefrontal cortex increased the level of mind-wandering. We also show that stimulation had no major effect on the level of meta-awareness of the attentional focus. Taken together, our results indicate that mind-wandering − probably the most internal and self-related mental function − can be modulated externally, that at least in some cases mind-wandering might not be regulated by meta-awareness, and that the frontal lobes might play a causal role in mind-wandering.

## Introduction

Mind-wandering is an intriguing and enigmatic phenomenon^1,2^. Most human behaviors and experiences are associated with processing and interacting with the external world around us. Mind-wandering, by contrast, is an internal experience that might be completely independent of the external world. Given the abundance of mind-wandering in our lives^3^, this mental function is likely to be essential^4,5^. Thus, understanding the cognitive and neural mechanisms of mind-wandering is of primary importance. At the neural level, functional MRI [fMRI] studies found that the default mode network^6–11^ as well as executive control network^6,8,12^ are activated during mind-wandering. However, fMRI studies are correlational by nature and therefore are unable to establish a causal link between behavior and neural substrate.

A recent study by the leading author of the present paper^13^ was the first to investigate mind-wandering in a causal way by using transcranial direct current stimulation (tDCS; non-invasive brain stimulation with a low electrical current)^14,15^. Axelrod and colleagues found that stimulation of the frontal lobes (anode: left dorsolateral prefrontal cortex; cathode: right supraorbital area) increased propensity to mind-wander. This suggested that: (a) mind-wandering can be modulated by means of external stimulation; and (b) the frontal lobes play a causal role in mind-wandering. The results of several more recent studies have generally been in line with these original findings. Specifically, it has been shown that cathodal stimulation of the frontal lobes (i.e., a reverse polarity to what was used in the study of Axelrod *et al*.) can decrease the propensity to mind-wandering^16–18^. Notably, these three recent studies were not a direct replication of the Axelrod *et al*. results because different behavioral paradigms and/or different stimulation procedures (e.g., tDCS montage) were used. Given the potential importance of the original findings of Axelrod and colleagues^19,20^, the widely-acknowledged problem of result replication in cognitive sciences^21^ and concerns regarding the reliability of the effects obtained in tDCS experiments^22^, it was important to replicate directly the results of the original study. In addition, to better understand the cognitive mechanisms of mind-wandering, it was of interest to examine whether the increase in mind-wandering as a result of stimulation was accompanied by a change in the meta-awareness of mind-wandering^19,20^. That is, what role meta-awareness plays in mind-wandering processing is not clear today^23^. Mind-wandering episodes can happen with or without a meta-awareness of them^1,23^. Performance of external tasks, such as the number of response inhibition failures^24^ and reader comprehension deficits^25^ can vary depending on whether participants mind-wandered with or without awareness during the experiment. From a theoretical point of view, meta-awareness might contribute to the regulation of mind-wandering^23^. Accordingly, when we stimulate the prefrontal cortex^13^, it is possible that stimulation alters the level of meta-awareness of mind-wandering, which in turn leads to an increased level of mind-wandering^19^. The original study of Axelrod and colleagues included only thought samples of task-unrelated thoughts (i.e., a proxy of mind-wandering); therefore, the role of meta-awareness could not be tested.

**Figure 1:**
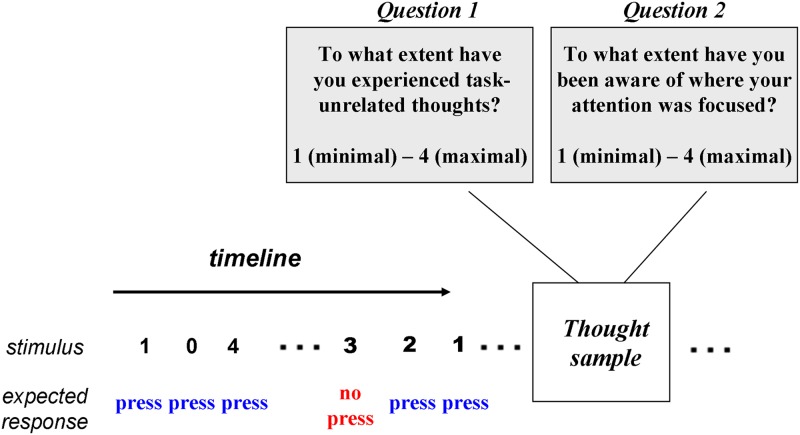
Experimental paradigm. A standard Sustained Attention to Response Task (SART) was used. Digits 0–9 appeared on the screen for 1 second. Interstimulus interval (ISI) is 1.2 seconds. Participants had to respond each time they detected a digit other than “3”. For digit “3” they refrained from responding. The level of task-unrelated thoughts (i.e., a proxy measure of mind-wandering) and the level of meta-awareness about mind-wandering were measured using periodic thought samples (Questions 1 and 2 on the figure).

In the present study, we examined the causal influence of non-invasive brain stimulation (tDCS) on mind-wandering. Our goal was twofold. First, we were interested in reproducing the original findings of Axelrod and colleagues^13^, showing that stimulation of the frontal lobes increases mind-wandering. To increase the reliability and the power of the replication, we used a larger sample size and ran the experiment in another country/language, operated by a different research experimenter. Our second goal was to examine whether the increase of mind-wandering as a result of stimulation was associated with a change in meta-awareness of mind-wandering. As a paradigm, we used the Sustained Attention to Response Task (SART)^26^ with periodic thought samples (i.e., two questions presented one after another; see Fig. 1). The design of the experiment was almost identical that of the previous study^13^, while the only major change was the addition of the thought sampling rating regarding meta-awareness of the attentional focus (see Fig. 1). Three groups of participants took part in the study (one visit per participant; between-subject design): anodal left dorsolateral prefrontal cortex [DLPFC] stimulation (N = 30); sham stimulation (N = 28; the same montage as the first group, but without actual stimulation); and anodal occipital cortex stimulation (control region; N = 27). In all three groups, the cathode electrode was placed at the right supraorbital area.

## Results

**Figure 2:**
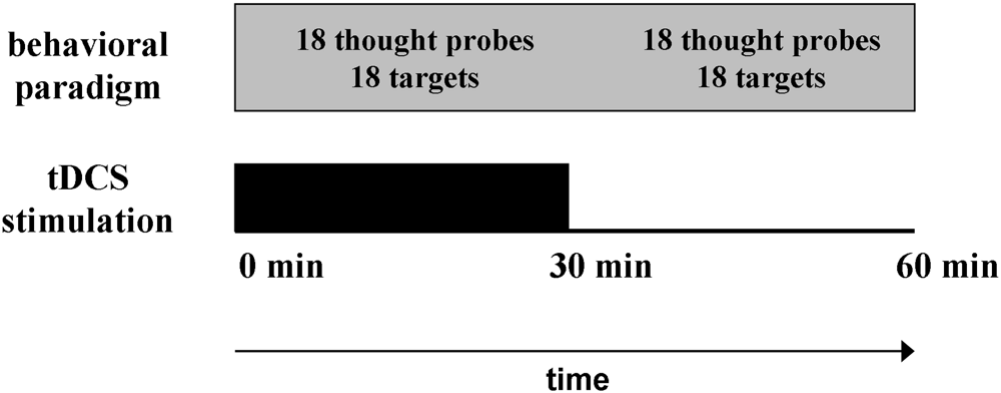
Stimulation protocol. Experimental paradigm and the stimulation started simultaneously. After 30 minutes, the stimulation ended (without the participants being aware of this). The experimental paradigm continued for additional 30 minutes. Each half of the experiment (30 minutes) contained 18 thought probes and 18 target stimuli.

Experimental paradigm duration was about 1 h, and the stimulation was applied during the first half of the experiment (Fig. 2). We refer to the first period as the “online stimulation” and to the second period as the “offline stimulation.” We first present the results of the full experiment (i.e., both parts together; 36 thought probes and 36 targets).

### Task-unrelated thoughts and meta-awareness (full experiment)

**Figure 3:**
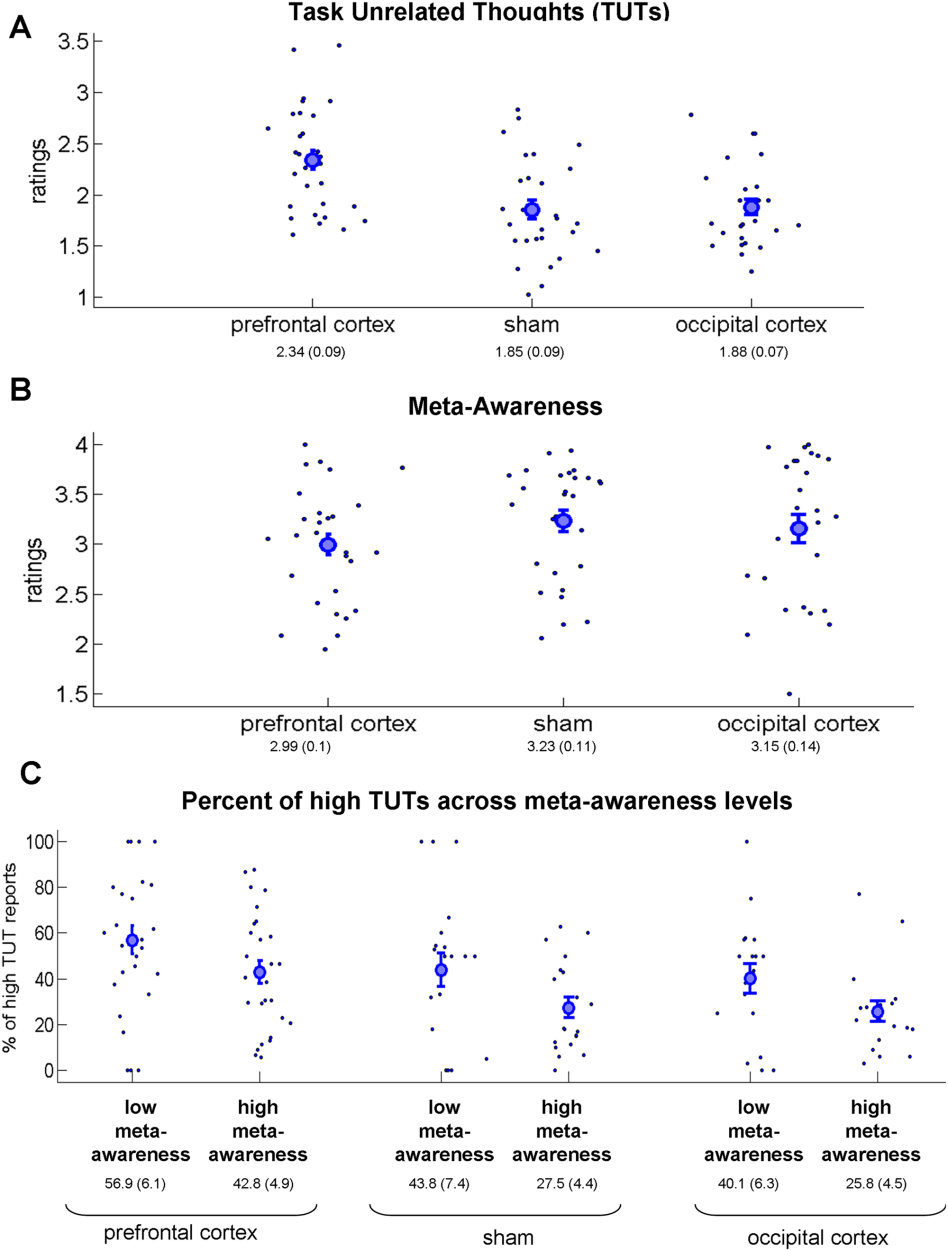
Results of mind-wandering and meta-awareness reports (full experiment, both online and offline stimulation). In all subfigures, the small dots represent the results for individual participants. The large circle is the group average, and the error bars reflect the standard error of mean. The three conditions are: stimulation of the prefrontal cortex, sham stimulation, and stimulation of the occipital cortex (control region). (**A**) Task-unrelated thought (TUT) responses. Ratings ranged from 1 (minimal TUT) to 4 (maximal TUT). Note that there were far more TUTs in frontal stimulation condition than for the sham and occipital cortex stimulation conditions. (**B**) Meta-awareness responses regarding mind-wandering. Ratings ranged from 1 (minimal meta-awareness) and 4 (maximal meta-awareness). No major difference was found between the conditions. (**C**) Percent of high-TUT reports (ratings 3−4) across two categories of meta-awareness [low level of meta-awareness (ratings 1 and 2) and high level of meta-awareness (ratings 3 and 4)]. Note that a) in all three conditions (irrespective of stimulation), the lower level of meta-awareness was associated with a higher percentage of high-TUT reports, and b) the stimulation did not change the relative relationship of high-TUT reports for two categories of meta-awareness.

Stimulation of the frontal lobes, but not sham or occipital cortex stimulation, increased the level of task-unrelated thoughts (TUT; Fig. 3A; the mean and SEM numbers are provided on the plots). Statistically, one-way ANOVA (three levels: prefrontal cortex stimulation, sham stimulation, and occipital cortex stimulation) revealed a strong significant main effect: *F*(2,82) = 9.77; *p* < 0.001; η^2^ = 0.19 (Bayes factor of 172 in Bayesian one-way ANOVA). Post-hoc two-sample two-tail *t*-tests showed that prefrontal cortex stimulation increased the TUT compared with the sham stimulation [*t*(56) = 3.66; *p* < 0.001; Cohen’s *d* = 0.97] and compared with the occipital cortex [*t*(55) = 3.75; *p* < 0.001; Cohen’s *d* = 1]. These results fully replicate those of the original report^13^.

We then tested whether stimulation affected meta-awareness of the attentional focus. We can see (Fig. 3B) that the prefrontal stimulation slightly decreased the level of meta-awareness. Statistically, we found no significant difference between conditions (one-way ANOVA main effect: *F*(2,82) = 1.11; *p* = 0.33; η^2^ = 0.026). Seeking stronger statistical support, we applied a Bayesian approach that is capable not only of rejecting H_0_ but also of supporting H_0_^27^. Using one-way Bayesian one-way ANOVA, we found a Bayes factor of 0.25, indicating substantial evidence in support of H_0_ (Bayes factor <0.33)^28^. Next, we examined the relationship between TUT and meta-awareness. For each participant in each of the two categories of meta-awareness [low level of meta-awareness (ratings 1 and 2) and high level of meta-awareness (ratings 3 and 4)], we extracted the percentage of trials that was reported as “high TUT” (ratings 3 and 4). Note that the “low TUT” is just a complementary value (“low TUT” = 100 − “high TUT”). Had the stimulation influenced TUT by means of changing meta-awareness, we could expect a relationship of TUT ratings for low and high meta-awareness categories to vary across stimulation conditions. The results of this analysis are shown in Fig. 3C. We can see that, in all three conditions (irrespective of stimulation), the lower level of meta-awareness was associated with a higher percentage of high TUT reports. Critically, we can see that the stimulation did not change the relative relationship of high TUT reports for two categories of meta-awareness. To examine the relationship between TUT and meta-awareness statistically, we conducted a mixed two-way ANOVA with the type of stimulation as a between-subject factor (three levels: prefrontal cortex stimulation, sham stimulation, and occipital cortex stimulation) and level of meta-awareness as a within-subject factor (low meta-awareness and high meta-awareness). We found the main effects of the stimulation type [*F*(2,62) = 4.16; *p* = 0.02; η^2^ = 0.12] and level of meta-awareness [*F*(1,62) = 13.4; *p* = < 0.001; η^2^ = 0.18] but no effect of interaction [*F*(2,62) < 1; η^2^ = 0]. To further examine the “interaction” effect, we conducted a Bayesian one-way ANOVA with type of stimulation as a factor, treating the difference between low and high meta-awareness values as a dependent variable. The main effect in this one-way ANOVA is equivalent to the interaction effect in the original two-way mixed ANOVA. The Bayes factor was 0.132, thus supporting H_0_ (i.e., no difference between conditions). Similar results were obtained when, instead of percentage of trials with high TUT in the analysis, we compared the average level of TUT per meta-awareness level. Thus, we conclude that the stimulation did not affect the relationship between mind-wandering and meta-awareness.

### External task execution (full experiment)

**Figure 4:**
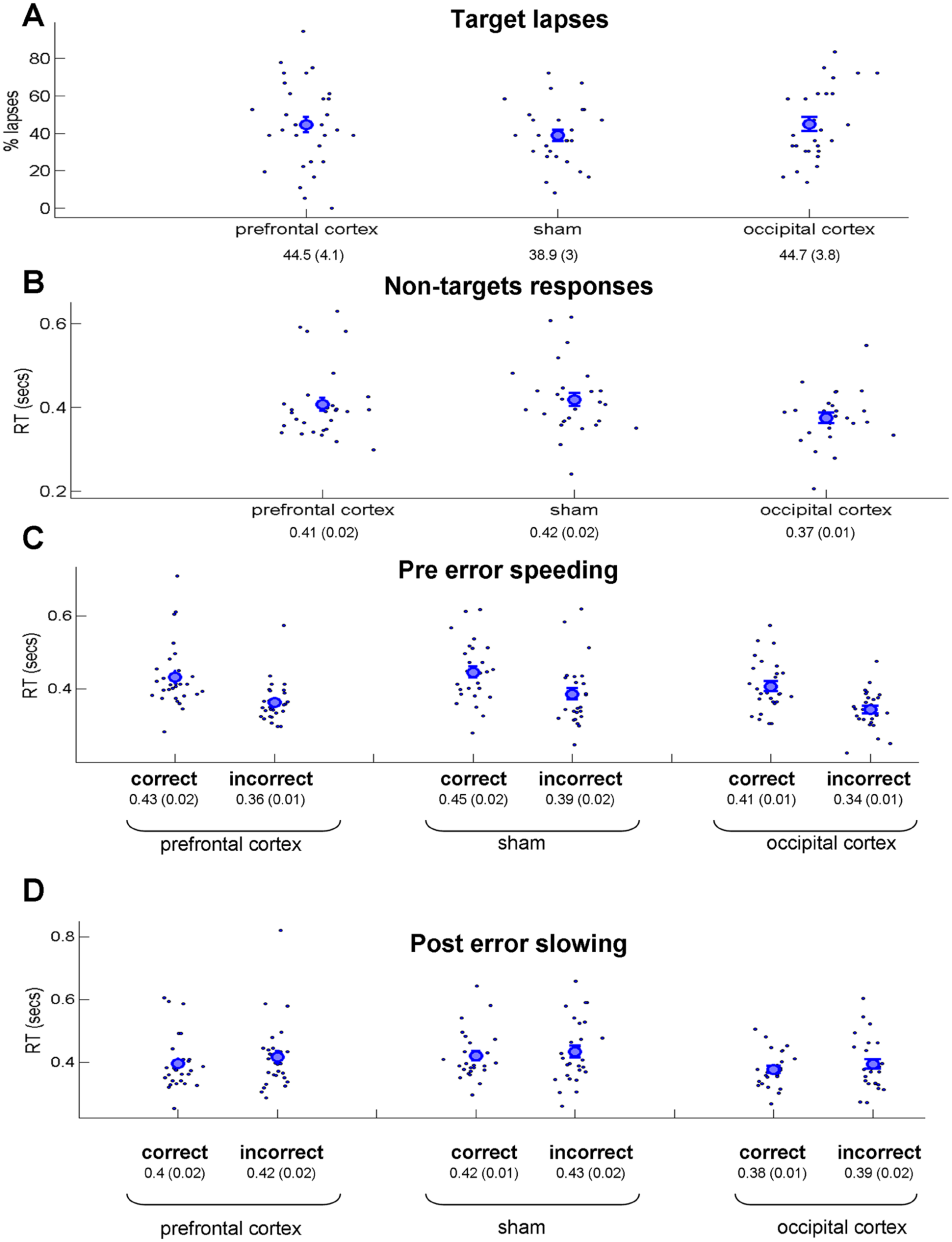
Results of the external task execution measures (full experiment, both online and offline stimulation). The same convention as in Fig. 3. (**A**) Percentage of target lapses (i.e., the cases when participants erroneously failed to refrain from the response for the target stimulus). (**B**) Response times for non-targets. (**C**) “Pre error speeding” results (i.e., faster non-target response rates prior to erroneously responding for the target stimulus). (**D**) “Post error slowing” (i.e., slower non-target response rates after erroneously responding for the target stimulus). In all four measures no major difference was found between the conditions.

Next, we examined whether stimulation had any effect on execution of the external task. A comparison of percentage of target lapses between conditions revealed no difference between conditions (Fig. 4A; one-way ANOVA: *F*(2,82) < 1; Bayesian one-way ANOVA Bayes factor of 0.194). Also, there was no major difference between conditions for the response time for non-target stimuli (Fig. 4B; one-way ANOVA: *F*(2,82) = 2.4; *p* = 0.1; η^2^ = 0.055; Bayesian one-way ANOVA Bayes factor of 0.675). Additionally, we examined “pre-error speeding” (i.e., faster non-target response rates prior to erroneously responding to the target stimulus) and “post-error slowing” (i.e., slower non-target response rates after erroneously responding to the target stimulus)^29^. In the pre-error speeding analysis, in all three conditions we found faster response rates for non-target trials that preceded incorrect target responses compared with correct target responses (Fig. 4C). Statistically, we conducted a mixed two-way ANOVA with type of stimulation between subject factors and correctness of target responses within subject factors (correct and incorrect). We found a strong main effect of the correctness of target response [*F*(1,81) = 119; *p* < 0.001; η^2^ = 0.59], but no main effect of stimulation type [*F*(2,81) = 2.45; *p* = 0.09; η^2^ = 0.057] and no effect of interaction [*F*(2,81) < 1; η^2^ = 0]. The Bayesian one-way ANOVA (difference between correct and incorrect target response values as dependent variables) revealed a Bayes factor of 0.106, thus supporting H_0_ (i.e., no difference between conditions). In the post-error speeding analysis, in all three conditions, we found slower response rates for non-target trials that followed incorrect target responses compared with correct target responses, although the effects were relatively small (Fig. 4D; potential reasons for relatively small effects are considered in the general discussion). Using a mixed two-way ANOVA, we found the main effect of the correctness of target response [*F*(1,81) = 4.27; *p* = 0.042; η^2^ = 0.05] but no main effect of stimulation type [*F*(2,81) = 2.01; *p* = 0.14; η^2^ = 0.047] and no effect of interaction [*F*(2,81) < 1; η^2^ = 0]. The Bayesian one-way ANOVA using the difference between correct and incorrect target response values as dependent variables resulted in a Bayes factor of 0.112, thus providing support for H_0_ (i.e., no difference between conditions). Thus, the results of various measures of execution of the external task demonstrate no differences among prefrontal cortex stimulation, sham stimulation, and occipital cortex stimulation.

### Relationship between TUT and external task execution (full experiment)

**Figure 5:**
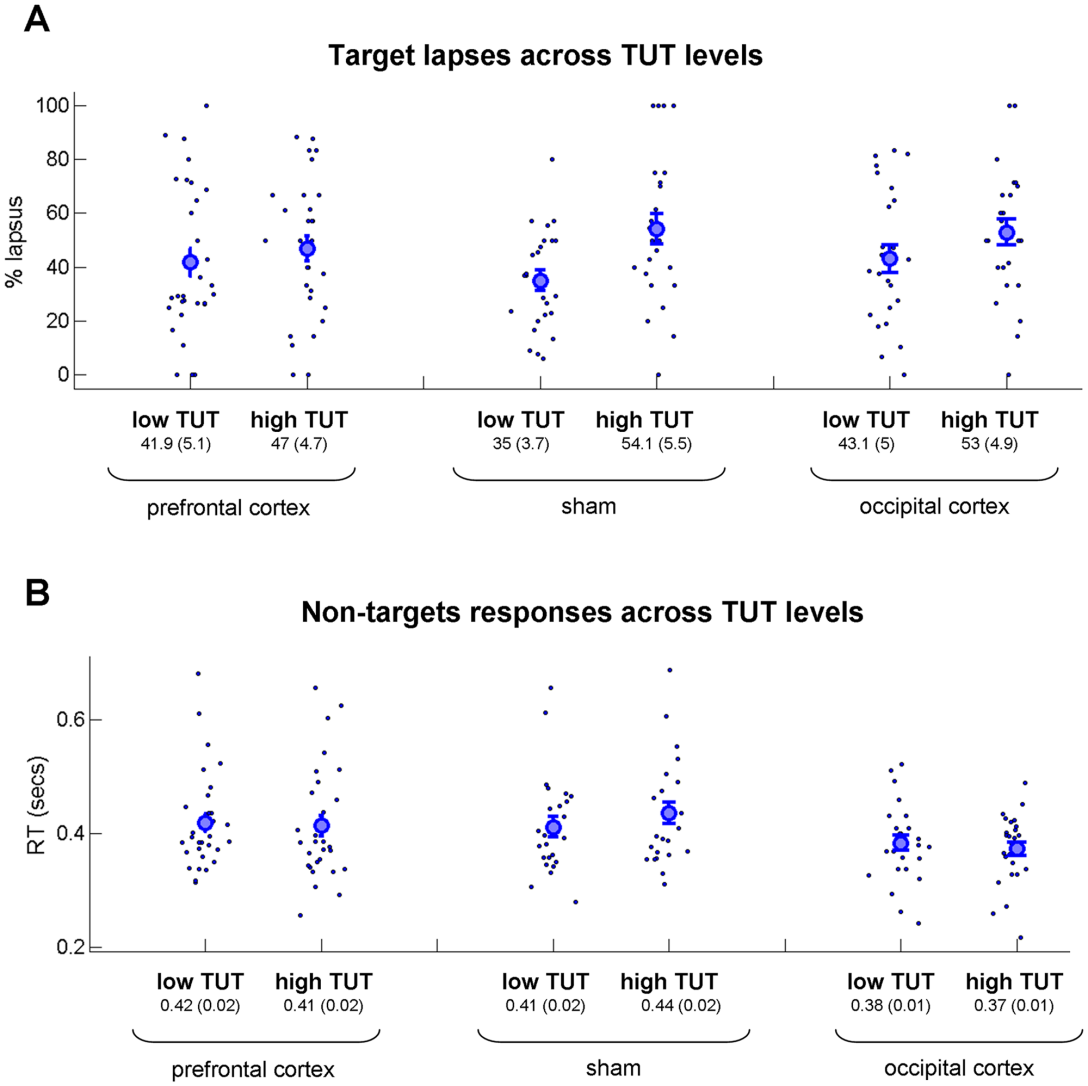
Target lapses and non-target response rates across TUT levels (high TUT, ratings 3−4 and low TUT, ratings 1−2; full experiment, both online and offline stimulation). (**A**) Percent of target lapses across TUT levels. Analysis included data segments of the experiment in which a non-target sequence that ended with a target trial was followed by a non-target sequence that ended with a probe trial. (**B**) Non-target response rates across TUT levels. Non-target trials were taken immediately prior to the probe question. The critical result was for both analyses no interaction was also found between stimulation conditions.

In addition, we asked: (a) whether there was a relationship between the level of TUT and the way the subjects performed the external task, and (b) whether the conditions differ with respect to this relationship. In our first analysis, we tested the relationship between percentage of target errors (i.e., lapses) and the level of TUT. To this extent, we identified segments of the experiment in which a non-target sequence that ended with a target trial was followed by a non-target sequence that ended with a probe trial (see Fig. 1 and Methods). In all three conditions, we found that the percentage of lapses was higher when the level of TUT was high (Fig. 5A). Statistically, we conducted a mixed two-way ANOVA with type of stimulation between subject factor and the level of TUT as within subject factor (low TUT and high TUT). We found a main effect of the level of TUT [*F*(1,77) = 11.8; *p* < 0.001; η^2^ = 0.13] but no main effect of stimulation type [*F*(2,77) < 1; η^2^ = 0] and no effect of interaction [*F*(2,77) = 1.55; *p* = 0.22, η^2^ = 0.03]. A Bayesian one-way ANOVA (difference between high and low TUT response values as dependent variables) yielded a Bayes factor of 0.365. In our second analysis, we tested the relationship between response time to non-target stimuli prior to the TUT probe and the level of TUT (see Methods). In all three conditions we found no major difference between response times for non-target stimuli between low and high TUT (Fig. 5B). Statistically, we conducted a mixed two-way ANOVA with type of stimulation as between subject factor and the level of TUT as within subject factor (low TUT and high TUT). Neither of the effects was significant in this analysis in terms of the main effect of the level of TUT [*F*(1,78) < 1; η^2^ = 0], main effect of stimulation type [*F*(2,78) = 2.58; *p* = 0.08; η^2^ = 0.06], and interaction [*F*(2,78) = 2.41; *p* = 0.1; η^2^ = 0.06]. The Bayesian one-way ANOVA (difference between high and low TUT response values as dependent variables) revealed a Bayes factor of 0.68. Note that the relationship between TUT level and the response time for non-target stimuli is not a well-established phenomenon (e.g., ref.^6^ did not find it, either). Taken together, using the measures of relationship between the level of TUT and the performance of the external task, we found no evidence that the three stimulation conditions are different.

### Comparison between online and offline stimulation

**Figure 6:**
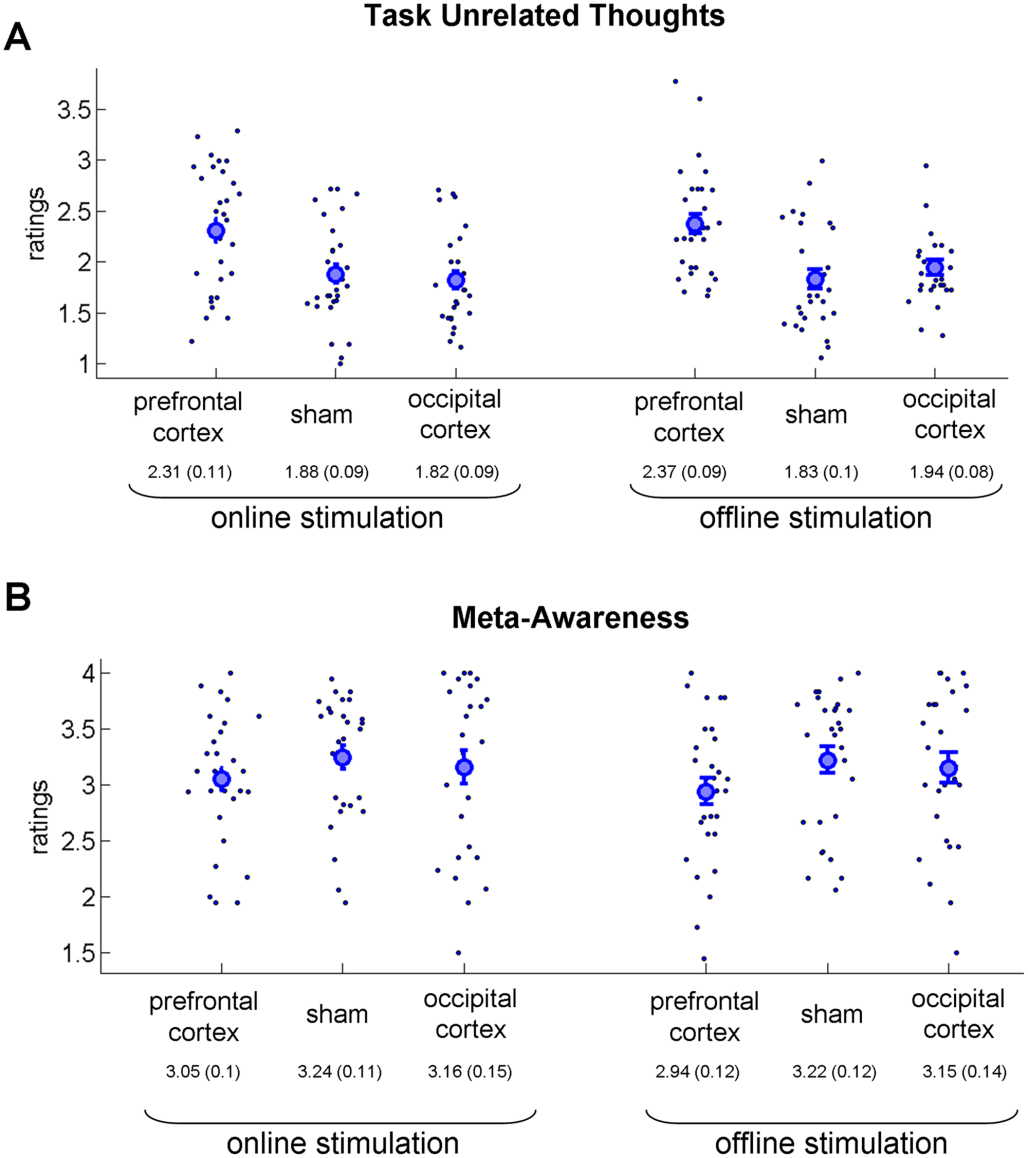
Results of mind-wandering and meta-awareness reports (comparison of online and offline stimulation parts of the experiment). Each part lasted about 30 minutes and included 18 thought probes and 18 target stimuli. The results of the online and offline stimulation are shown at the left and right sides, respectively. The large circle is the group average, and the error bars reflect standard error of mean. The results of all three measures did not differ between the online and offline stimulation parts. (**A**) Task-unrelated thought (TUT) responses. (**B**) Meta-awareness responses regarding mind-wandering.

**Figure 7:**
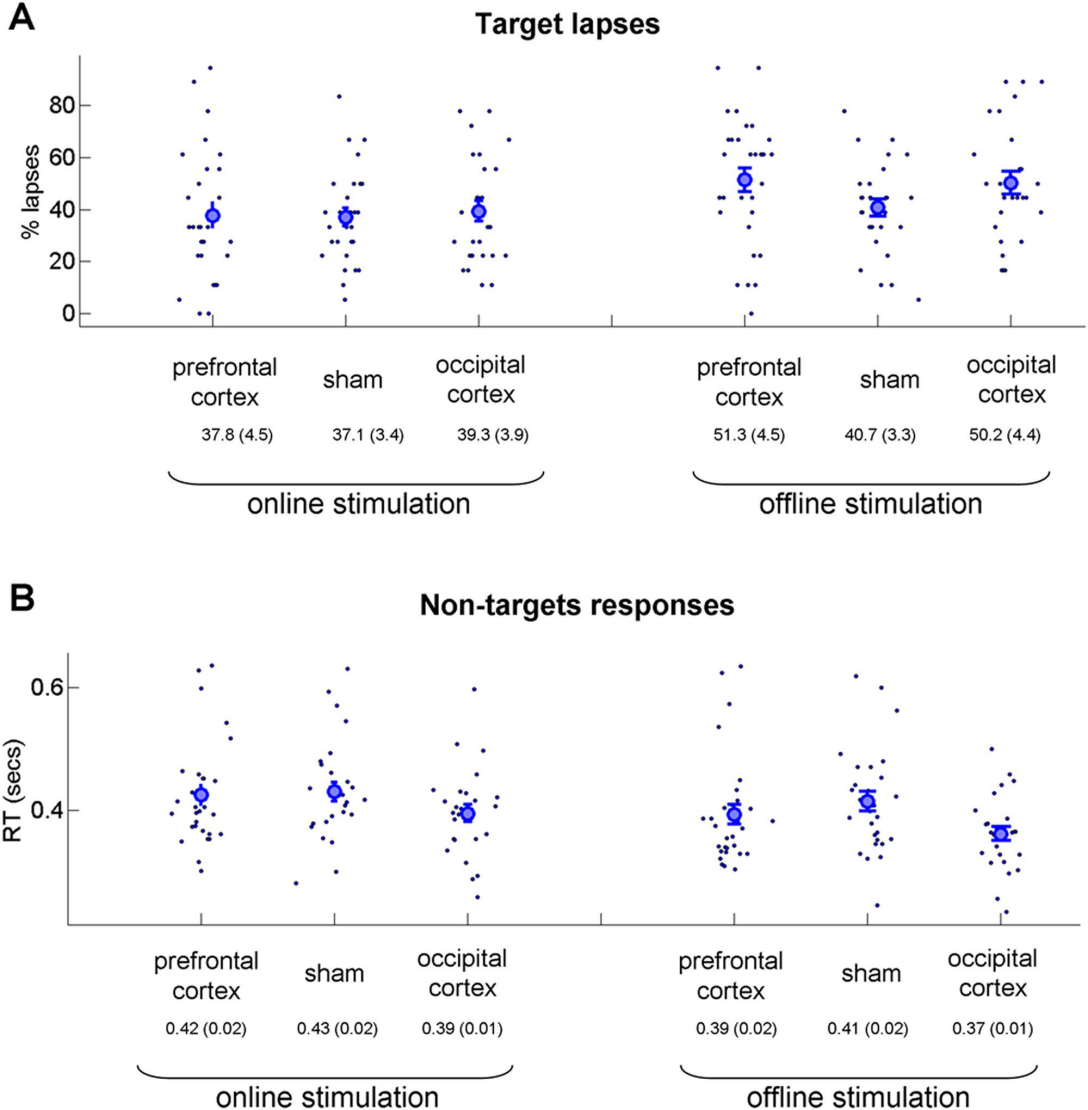
Results of the external task execution measures (comparison of online and offline stimulation parts of the experiment). (**A**) Percentage of target lapses (i.e., the cases when participants erroneously failed to refrain from the response for the target stimulus). (**B**) Response times for non-targets. In both measures no major difference was found between the conditions (i.e., no statistical interaction).

Physiological mechanisms of online (i.e., the experimental paradigm in parallel to the stimulation) and offline (i.e., the experimental paradigm after the stimulation) stimulation have been suggested to differ^30^. Thus, it was of interest to compare the behavioral effects of offline and online stimulation. For each of the measures, we conducted mixed two-way ANOVA with type of stimulation as between subject factors (three levels: prefrontal cortex stimulation, sham stimulation, and occipital cortex stimulation) and stimulation protocol as within subject factor (online stimulation, offline stimulation). For the level of TUT (Fig. 6A), in line with the analysis of the full experiment, we found a strong main effect of the stimulation type [*F*(2,82) = 9.79; *p* < 0.001; η^2^ = 0.19] but no main effect of the stimulation protocol [*F*(1,82) = 1.07; *p* = 0.3, η^2^ = 0.12] or interaction [*F*(2,82) = 1.29; *p* = 0.27; η^2^ = 0.03]. To further examine the “interaction” effect, we conducted a Bayesian one-way ANOVA with three factors (prefrontal cortex stimulation, sham stimulation, and occipital cortex stimulation) and the difference between online and offline stimulation values as dependent variables. Bayes factor in one-way Bayesian ANOVA was 0.286. For meta-awareness (Fig. 6B), the mixed two-way ANOVA revealed no significant effects: main effect of stimulation type [*F*(2,82) = 1.09; *p* = 0.33; η^2^ = 0.026], main effect of stimulation protocol [*F*(1,82) = 1.33; *p* = 0.25; η^2^ = 0.016], and interaction [*F*(2,82) < 1]. Bayesian one-way ANOVA of differences revealed a Bayes factor of 0.161. With regard to performance of the external task, we first examined the percentage of target lapses (Fig. 7A). Mixed two-way ANOVA revealed a significant effect in terms of the part of the experiment [*F*(1,82) = 24.09; *p* < 0.001; η^2^ = 0.22] but no main effect of stimulation type [*F*(2,82) < 1] and no significant interaction [*F*(2,82) = 2.48; *p* = 0.08; η^2^ = 0.044]. Bayesian one-way ANOVA of differences (i.e., equivalent to interaction in the two-way ANOVA) found a Bayes factor of 0.723. In addition, we examined the response time for non-target stimuli (Fig. 7B). The mixed two-way ANOVA revealed a significant effect in terms of part of the experiment [*F*(1,82) = 37; *p* < 0.001; η^2^ = 0.3] but no main effect of stimulation type [*F*(2,82) = 2.29; *p* = 0.1; η^2^ = 0.05] and no significant interaction [*F*(2,82) = 1.27; *p* = 0.28; η^2^ = 0.02]. The Bayesian one-way ANOVA of differences (i.e., equivalent to interaction in the two-way ANOVA) found a Bayes factor of 0.282. Overall, for all tested measures, we found no interaction between stimulation condition (i.e., prefrontal cortex/sham/occipital cortex stimulation) and stimulation protocol (online/offline stimulation). Also, corresponding Bayes factors in ANOVAs for all measures were low. Thus, we conclude that, at least in our experiment, the effects of online and offline stimulation did not differ.

## Discussion

In the present study, we successfully replicated the early results of Axelrod and colleagues^13^ by showing that stimulation of the frontal lobes, but not sham stimulation or stimulation of the control region, increases the propensity to mind wandering. Stimulation of the frontal lobes had no major impact on the level of meta-awareness of mind-wandering, and the increase in the level of mind-wandering as result of stimulation was unrelated to the meta-awareness level. These and additional findings are discussed in detail below.

Mind-wandering is one of the most fascinating capacities of our brain and mind. At any moment in time, we can disconnect from the external world and have a rich virtual experience within us. But in real life, it is our brain that initiates whether to switch to a mind-wandering state. An early report of the leading author of the present study^13^ was the first to show that it is also possible to modulate mind-wandering externally by means of electrical stimulation. This result emphasized that even very-internal and self-related mental functions like mind-wandering can be modulated externally by merely changing the neural electric field. An additional benefit of stimulation methods is that they permit establishing a functional role of a neural substrate in a causal role. That is, fMRI studies have implicated various parts of the frontal lobes in mind-wandering^11,12,31^, but fMRI methodology is only correlational. The study of Axelrod and colleagues^13^ was the first to demonstrate that frontal lobes are causally involved in mind-wandering. Both findings, that it is possible to increase mind-wandering using stimulation and that the frontal lobes are causally involved in mind-wandering, were suggested to be important^19,20^. Accordingly, it was essential to reproduce them, and this was one of the goals of the present study. We fully accomplished this goal by demonstrating that stimulation of the frontal lobes, but not sham or occipital cortex control region stimulation, increases the propensity to mind-wander. The effect sizes in our study were large (Figs 3A and 6A). To increase the reliability and power of our replication: a) we used a larger sample size that was more than twice that of the original sample size; b) we conducted the experiment in a different country and language (China/Chinese language instead of Israel/Hebrew language); and c) the experiment was conducted by a different research experimenter (X.Z. instead of V.A. in the original study), and as such, even if experimental bias was inadvertently introduced in the original experiment, this bias was not repeated in the replication. Overall, the fact that the key effect was replicated increases the likelihood that the effect is genuine.

Since the original report^13^, several studies have explored mind-wandering using tDCS stimulation of the frontal lobes. Kajimura, Nomura and colleagues^16,17^ conducted two studies using a visual search paradigm with periodic thought samples. The authors found that cathodal stimulation of the left frontal lobes (cathode: left DLPFC; anode: right inferior parietal lobule) decreased the level of mind-wandering compared to the reversed polarity montage. In an additional study, Bertossi and colleagues^18^ used a sustained attention task similar to what we used here. The authors found that cathodal stimulation of the frontal lobes (Fpz region according to 10–20 EEG; cathode at the deltoid muscle) reduced mind-wandering in males. Note that compared to our study, all the aforementioned studies used reversed polarity (i.e., cathode electrode at the frontal lobes instead of anode electrode at the frontal lobes, as was used in our study). Anodal tDCS stimulation is thought to increase cortical excitability, while cathodal tDCS stimulation is thought to decrease cortical excitability^32,33^. Accordingly, it is reasonable that the behavioral effects of a stimulation with opposite polarity will also be opposite^34^. It is noteworthy that while all discussed tDCS studies (including our two studies) stimulated the frontal lobes, the tDCS montage of these studies varied substantially, especially the location of the non-frontal electrode. Nevertheless, in all five studies the stimulation of the frontal lobes modulated mind-wandering. Thus, the accumulated evidence supports the notion of the important role that the frontal lobes play in mind-wandering. This conclusion is further supported by a recent neuropsychological study that found that patients with lesions of the ventromedial prefrontal cortex also experience reduced mind-wandering^35^.

Non-invasive stimulation tools and tDCS in particular have a relatively limited spatial resolution^30,36^. Accordingly, the goal of the present and the original study^13^ was not to establish precisely which of the frontal lobe’s sub-regions were involved in mind-wandering. As previously discussed in the original paper^13^, given the montage that we used (anode: left DLPFC, cathode: right supraorbital area), there are three most plausible candidate frontal lobe loci to being stimulated. First, there is the left DLPFC, the region previously implicated in mind-wandering^6,8,12^. The DLPFC was proximate to the anodal electrode. Accordingly, given that the strongest current is induced at cortical locations that are most proximate to the electrodes^14,37^, the DLPFC was likely stimulated. Second, there is the medial frontal region, which has also been previously implicated in mind-wandering^6–11,35^ and self-referential and self-related processing^38–41^. In addition, two combined tDCS-fMRI studies demonstrated that stimulation of the frontal lobes (the same montage as we used) could potentially induce changes in the functional connectivity of the medial frontal regions^42,43^. The third candidate is the frontopolar cortex, a region under the cathode electrode that could also be stimulated^14,36,44^. This region has been implicated previously in self-generated processing^45^. Thus, each of these regions separately or together could potentially contribute to increased levels of mind-wandering. In the future, more precise localization can be potentially achieved using a combined tDCS-fMRI approach^42,43^.

In addition to mind-wandering thought samples, the present study also included thought samples of meta-awareness of mind-wandering. Mind-wandering episodes can happen with or without meta-awareness^1,23^. From a theoretical perspective, meta-awareness might contribute to the regulation of mind-wandering^23^. Thus, it was of interest whether stimulation of the frontal lobes in addition to mind-wandering altered the level of meta-awareness, and whether the change in mind-wandering level as a result of stimulation was related to a change in meta-awareness^19,20^. We found a slight decrease of meta-awareness as a result of frontal lobe stimulation. Both Pearson, and critically, Bayesian ANOVA established that there was no difference in meta-awareness between stimulation conditions. We also found that as a result of stimulation, the percentage of mind-wandering reports increased both for low and high levels of meta-awareness, and that the relationship between mind-wandering and meta-awareness did not differ between conditions. Thus, our overall results show that at least in some cases, changes in mind-wandering are likely not mediated by or regulated by meta-awareness^23^.

In the present study, we adopted a widely-used approach to test task-unrelated thoughts and meta-awareness about attentional focus^6^. The two-probe thought questions in this paradigm regarding the level of TUT and meta-awareness appear in a fixed and constant order. The benefit of this fixed order is that in the course of the experiment, the participant becomes used to a single order. As a result, introspective reports can be provided with little attention and resources devoted to instructions. In addition, for the present experiment, it was also important to have the mind-wandering report at the first position because we aimed to replicate the study in which the mind-wandering report was the only report. Having said that, it should be acknowledged that the fixed order of probe questions might also have some downsides. For example, because the meta-awareness report always appears second, the meta-awareness report might suffer from a less vivid memory regarding what happened before the probe (i.e., a less reliable meta-awareness report). In addition, the fact that the meta-awareness report always appears after the mind-wandering report might also to some extent bias the decision. That is, the mere fact that the participant just reported that they mind-wandered might potentially bias the report about meta-awareness. Thus, in the future, it would be of interest to explore whether similar results are obtained when probe questions appear in a counterbalanced order. In a similar vein, the mere presence of the probe report about mind-wandering, regardless of its position, might potentially bias the results of the meta-awareness report. Thus, in the future, it might be of interest to validate the results of meta-awareness reports when there is no mind-wandering report at all (i.e., a probe with a single question). Finally, it should be noted that it has been recently suggested that an important dimension of mind-wandering is its intentionality^46,47^. In the present study we did not test this dimension, so in the future it might be of interest to examine whether stimulation of the frontal lobes might modulate the intentionality of mind-wandering.

In addition to examining introspective probe ratings (i.e., mind-wandering and meta-awareness), we also tested external task execution measures. In line with previous literature^29^, we found a “pre-error speeding” effect (i.e., faster non-target response rates prior to erroneously responding to the target stimulus; Fig. 4C). Interestingly, the analogous “post-error slowing” effect (i.e., slower non-target response rates after erroneously responding to the target stimulus) was relatively weak (Fig. 4D). However, it should be kept in mind that in our experiment, the target trial always appeared after at least ten non-target trials. Thus, after committing an error, the participants knew that the target trial was not going to appear in foreseeable future (therefore, there is no reason to slow down their response rate). In addition, our task was very monotonous with a slow stimulus presentation rate (i.e., a stimulus every 2.2 sec). For some trials, the participants might not even have been aware that they missed the target. In such a case, the slow-down effect might be absent or at least be different^48^. Most importantly, with regard to all tested external task execution measures, we did not find considerable differences between stimulation conditions. This result was obtained using both a Pearson (classical) ANOVA and a Bayesian ANOVA (Bayes factor). Apart from “pre-error speeding” and “post-error slowing” measures, of particular interest was the measure of percent of target response lapses, given that in the original study of Axelrod and colleagues^13^, the stimulation of the frontal lobes not only strongly increased mind-wandering, but also slightly (and not significantly) decreased the percentage of target lapses. Axelrod and colleagues previously suggested^13^ that brain stimulation might cause an enhancement (extension) of cognitive capacity, so that mind-wandering is increased without equally compromising the performance of the external task. In the present study, frontal lobe stimulation did not decrease, but slightly (not significantly) increased the percentage of target lapses. Thus, the present results do not support the original claims. Notably, the original study emphasized that the found effects were relatively weak and did not reach statistical significance in either of the two experiments. To this extent, the fact that the less strong effects of the original study could not be replicated underscores the importance of replicating procedures^21^. Future research, preferably with an even larger sample size, is needed for a better understanding of the relationship between mind-wandering and external task performance as a result of stimulation.

Finally, the design of our study permitted comparison between online and offline stimulation. In line with the original study^13^, we found no difference between the two types of stimulation. This result might be at odds with the notion that physiological mechanisms of online and offline might differ^30^; online anodal stimulation is thought mainly to change the resting membrane potential without inducing synaptic changes, while offline anodal stimulation is believed to modulate GABA and glutamate synapses^30^. However, as also discussed in detail in the original study^13^, empirical evidence as to the difference between the two types of stimulation is mixed. For example, in motor-learning paradigms, online stimulation effects were stronger [montage: motor cortex (anode), contralateral supraorbital ridge (cathode)^49^; montage: left F3 electrode site (anode), right upper arm (cathode)^50^]. In contrast, in one experiment using a visual perceptual learning task the offline stimulation effects were stronger [montage: the occipital cortex (anode), the right arm (cathode)^51^]. Future investigation of the biophysics of tDCS might be needed to better understand the divergence between the effects of offline and online stimulation. It should also be acknowledged that the design we used in our study might not be ideal for reaching a definitive conclusion regarding the difference between online and offline stimulation. That is, for all participants, the online and offline stimulation coincided with the first and second parts of the experiment, respectively. Accordingly, the parts of online and offline stimulation differed not only with regard to stimulation type. For example, in the second half of the experiment we found a greater number of target lapses, and faster response rates for non-targets. These changes might reflect greater fatigue and/or more automatic task execution during the second part of the experiment. While the changes between the two parts of the experiment were similar for all three stimulation conditions (i.e., no interaction with condition type), it was still a limitation. A better possible experimental design might compare offline and online stimulation between participants, for example: one group of participants will execute the task only during online stimulation, and the other group will first undergo stimulation without any task, and only once stimulation ends will start the experiment. In such a design, the two groups differ only with regard to stimulation protocol, but not the stage of the experimental paradigm. Note, that the limitation discussed above is only related to a comparison between the effects of online and offline stimulation.

In summary, the present study replicated an early report^13^ demonstrating that non-invasive stimulation of the frontal lobes can increase the propensity to mind-wander. In addition, the study found that the increase in the level of mind-wandering as result of stimulation was unrelated to meta-awareness level.

## Methods

### Participants

Ninety-one participants took part in the experiment. The experiment was approved by the Southwest University Brain Imaging Center Institutional Review Board. The experiment was conducted in accordance with institutional guidelines and regulations. All participants signed written informed consent forms before beginning the experiment. The data of six participants were excluded from the analysis due to the inability of participants to follow the instructions. Participants were randomly assigned to one of three groups: prefrontal cortex stimulation, sham stimulation, or occipital cortex stimulation. The number of participants in each group after exclusion of participants who did not follow the instructions was as follows: prefrontal cortex stimulation (*N* = 30; 12 males; mean age: 19.95); sham stimulation (*N* = 28; 11 males; mean age: 19.48); and occipital cortex stimulation (*N* = 27; 11 males; mean age: 19.79). The sample size (i.e., number of participants in each group) was determined *a priori* (i.e., before the experiment) using the G*Power 3 tool^52^. Specifically, the effect sizes of our previous tDCS study^13^ for comparison of two groups (i.e., two-samples tests) were about Cohen’s *d* = 1. For the present study, we required an alpha level of 0.01 and power of 0.8. In a two-tail test, this resulted in 26 participants per group (after exclusion). Given that we had not known ahead of time how many participants would be excluded due to the inability to follow the instructions, the actual number of participants slightly deviates from what we planned.

### Experimental apparatus, design, and stimuli

One of the central goals of the present study was to replicate the results of the study of one of the authors of the present research^13^. Accordingly, most of the procedures were deliberately matched to those used in the original study. The experiment was run in MATLAB 2010A with Psychtoolbox^53^. We used Sustained Attention to Response Task (SART) paradigm^6,26^ (see Fig. 1). A stream of digits from 0 to 9 in a pseudo-random order was shown on the screen. The color of the stimuli was black, and the background was grey (RGB: 104, 104, 104). The duration of the stimulus was 1 second, and the interstimulus interval (ISI) was 1.2 seconds. The task was to press on a spacebar as soon as a digit (“0”–“9,” except for “3”) appeared on the screen (i.e., non-target stimuli). Participants had to refrain from responding when digit “3” appeared (i.e., target stimulus). In addition, from time to time, the participants were presented with thought probes that included two questions, one after the other. The first question was “To what extent have you experienced task-unrelated thoughts?” The answer, according to a Likert scale, was: 1 (minimal) – 4 (maximal). The second question was “To what extent have you been aware of where your attention was focused?” The answer, according to a Likert scale, was: 1 (minimal) – 4 (maximal). The questions were in Chinese. The thought probe questions were in accordance with the study that we aimed to replicate^13^, along with the seminal study of Christoff and colleagues^6^. We ensured that participants clearly understood the questions. The target stimuli and thought probes appeared after a long sequence of non-target stimuli: average number of non-targets before target stimuli was 19.88 (std: 5.08; minimum 10; maximum 32), and average number of non-targets before thought probes was 18.33 (std: 4.9; minimum 11; maximum 32). Targets and thought probe sequences appeared in a pseudo-random order (the same order for all participants). The experiment was divided into six sessions, with six thought probes and six target stimuli in each session. The duration of the sessions was 9.37, 9.01, 9.26, 9.92, 9.74, and 8.57 minutes. No break nor rest was given between the sessions. The total duration of the experiment was close to 1 hour. Before the experiment, the participants underwent a short training session that included four targets and thought probes.

### Brain stimulation

The procedures were similar to those used in the study by Axelrod and colleagues^13^. A constant current tDCS stimulator was used (Soterix Medical). A saline-soaked pair of surface sponge electrodes (7 × 5 cm) was used. In the real stimulation condition, the current was gradually increased to 1 mA over 30 seconds, remained at 1 mA for 30 minutes, and was gradually decreased to zero over 30 seconds. In the sham stimulation condition, the procedure was similar, while the stimulation continued for only 2 minutes. The participants did not feel the current and were not aware of whether or not they were stimulated. In the prefrontal cortex stimulation condition, the anode was positioned at F3 (EEG 10–20 system), and the cathode at the right supraorbital area. In the sham stimulation, the same montage was used but without actual stimulation (see above). In the occipital cortex stimulation condition, the anode electrode was at the Oz position, and the cathode was at the Cz position. The stimulation started with the experimental paradigm (Fig. 2) and continued for 30 minutes (i.e., online stimulation). After that, the paradigm was run without stimulation (i.e., offline stimulation). For all participants, the experiment always started with stimulation (or sham stimulation). There was no counterbalancing of stimulation across participants. Counterbalancing stimulation would have created two completely nonequivalent sub-conditions: (1) starting with stimulation would have resulted in 30 minutes of online stimulation and then 30 minutes of offline stimulation (aftereffect of stimulation); (2) starting without stimulation would have resulted in 30 minutes of no stimulation at all, and then 30 minutes of online stimulation.

### Data analysis

Data analysis was conducted in MATLAB R2009B using custom code^54^. The data were analyzed using parametric tests (i.e., analysis of variance and *t*-tests). The normality assumption was tested using the Lilliefors test. In all cases, the distribution was normal or very close to normal (Lilliefors test, *p* > 0.05). To validate, all the analyses were also performed using non-parametric counterparts (Kruskal–Wallis one-way analysis of variance and Wilcoxon rank–sum tests); the results were similar to those when using parametric statistics. The analysis of the full experiment (Figs 3−5) was conducted based on the all data (36 data points per participant, thought sample, or target). The comparison between online and offline stimulation (Figs 6 and 7) was conducted based on 18 data points for each part. To examine the relationship between TUT and meta-awareness (Fig. 3C), we compared the percentage of high level TUT reports (ratings 3 and 4) for trials with a low level of meta-awareness (ratings 1 and 2) versus trials with a high level of meta-awareness (ratings 3 and 4). Only the data of participants who had both low and high meta-awareness ratings could be included (prefrontal cortex stimulation: *N* = 27; sham stimulation: *N* = 20; and occipital cortex stimulation: *N* = 18). Similar results were obtained if, instead of percentage of trials with high TUT in the analysis, we compared the average level of TUT per meta-awareness level. Analysis of the “pre-error speeding” (i.e., faster non-target response rates prior to erroneously responding to the target stimulus; Fig. 4C) and “post-error slowing” (i.e., slower non-target response rates after erroneously responding to the target stimulus; Fig. 4D) has been done in accordance with ref.^29^. Specifically, we extracted the non-target response rate of the trial that immediately preceded (“pre-error speeding”) or followed (“post-error slowing”) the target trial. The comparison was done between correct target responses (i.e., refraining from response to digit “3”) and incorrect target responses (i.e., no refraining from response to digit “3”). For the analysis testing the relationship between percentage of target errors (i.e., lapses) and the level of TUT (Fig. 5A), we identified segments of the experiment in which a non-target sequence that ended with a target trial was followed by a non-target sequence that ended with a probe trial. There were 23 such segments (two types of sequences appeared in a pseudorandom order; therefore, only part of the data could be used for this analysis). The correct and incorrect responses on targets (i.e., percent of lapses) were binned as to whether they were followed by low or high TUT reports. For the analysis testing the relationship between response time for non-targets and the level of TUT (Fig. 5B), we extracted response times of last-target trials immediately prior to the TUT probe (i.e., similar to what was done in “pre-error speeding” and “post-error slowing” analyses). We did not examine the relationship between “pre-error speeding”/“post-error slowing” and the level of TUT because we did not have sufficient data for a reliable analysis (i.e., on the one hand, only the segments of non-target-probe sequences could be used; on other hand, the participants had to have both low and high TUT trials). Bayesian analysis was conducted using the JASP package (https://jasp-stats.org/). In Bayesian ANOVA, the priors are based on Cauchy distribution^55^.

## Data Availability

The data supporting the findings of this study are available from the corresponding author upon reasonable request.
